# Supplementary material for: Implementing genomic screening in diverse populations
Source: Genome Med. 2021 Feb 5;13:17. doi: 10.1186/s13073-021-00832-y (PMC7863616; doi:10.1186/s13073-021-00832-y)
Supplement: Supplementary file 1 — Additional file 1: Table S1. Questions from a return of genomic results preference survey administered to previously enrolled BioMe Biobank participants. Table S2. Demographic characteristics of BioMe participants who were respondents of a return of genomic results preference survey (N = 72), and characteristics of all adult BioMe participants enrolled at the time of the survey (N = 31213). Table S3. Clinically confirmed pathogenic, likely pathogenic, and downgraded variants in a pilot genomic screening program. Fig. S1. A model for returning genomic results to biobank research participants without prior knowledge of genomic risk. Fig. S2. Pre-pilot survey to understand BioMe participants’ (N = 72) preferences regarding the hypothetical return of results. [file 13073_2021_832_MOESM1_ESM.pdf]

ADDITIONAL FILE 1

Implementing genomic screening in diverse populations

Noura S. Abul-Husn[1,2,3*], Emily R. Soper[1,2], Giovanna T. Braganza[1], Jessica E. Rodriguez[1], Natasha Zeid[4], Sinead Cullina[1], Dean Bobo[1], Arden Moscati[1,5], Amanda Merkelson[5], Ruth J.F. Loos[5], Judy H. Cho[2,3,5], Gillian M. Belbin[1,2], Sabrina A. Suckiel[1,2], Eimear E. Kenny[1,2,3]

[1]The Institute for Genomic Health, Icahn School of Medicine at Mount Sinai, New York, NY
[2]Department of Medicine, Icahn School of Medicine at Mount Sinai, New York, NY
[3]Department of Genetics and Genomic Sciences, Icahn School of Medicine at Mount Sinai, New York, NY
[4]Cardiogenetics, GeneDx Inc, Gaithersburg, MD
[5]The Charles Bronfman Institute for Personalized Medicine, Icahn School of Medicine at Mount Sinai, New York, NY

***Correspondence:** noura.abul-husn@mssm.edu

Supplementary Tables:

1. **Table S1.** Questions from a return of genomic results preference survey administered to previously enrolled Bio*Me* Biobank participants.

2. **Table S2.** Demographic characteristics of Bio*Me* participants who were respondents of a return of genomic results preference survey (N = 72), and characteristics of all adult Bio*Me* participants enrolled at the time of the survey (N = 31,213).

3. **Table S3.** Clinically confirmed pathogenic, likely pathogenic, and downgraded variants in a pilot genomic screening program.

Supplementary Figures:

1. **Fig. S1. A model for returning genomic results to biobank research participants without prior knowledge of genomic risk.** After genomics-first identification of variant positive individuals, they receive genetic counseling and are then referred to specialty provider(s) for further evaluation and initiation of risk management.

2. **Fig. S2. Pre-pilot survey to understand Bio*Me* participants' (N = 72) preferences regarding the hypothetical return of results. A)** Decision to enroll into biobank research if the program returned genomic results. **B)** Selected reasons for receiving or not receiving genomic results. **C)** Selected categories of genomic results participants would want to receive.

Table S1. Questions from a return of genomic results preference survey administered to previously enrolled Bio*Me* Biobank participants.

Question	Answer options
If, during your enrollment in *BioMe*, the recruiter told you that you **COULD** receive genetic research results from your participation, do you think you still would have enrolled?	• Definitely yes<br>• Probably yes<br>• Not sure<br>• Probably not<br>• Definitely not
What do you think are good reasons to receive genetic research results? (Check all that apply)	• To help myself (for example, by finding out what diseases I am at risk for)<br>• To help my family (for example, by finding out what diseases could run in the family)<br>• To help me make decisions about having children (for example, by finding out what diseases could be passed on to them)<br>• I feel ownership of my genetic research results<br>• Other (please specify)
What do you think are good reasons **NOT** to receive genetic research results? (Check all that apply)	• Concerns about my privacy<br>• Concerns about discrimination (health insurance, life insurance, employment, or other)<br>• Cannot make health changes based on genetic research results<br>• Receiving genetic research results would cause me or my family anxiety<br>• Other (please specify)
If given the choice, what type of genetic research results would you want to receive?	• Only genetic research results that the researcher thinks are important<br>• Only genetic research results about specific diseases that I think are important<br>• Only genetic research results about specific diseases that experts in genetics think are important and should be received by everyone<br>• All genetic research results, including those that are uncertain and/or cannot be interpreted at the moment<br>• None (I would not want to receive genetic research results)
If you were to receive genetic research results, how would you want to be informed?	• By phone<br>• By a letter in the mail<br>• In person<br>• Not applicable (I would not want to receive genetic research results)
If you were to receive genetic research results, who would you want to inform you?	• My primary care doctor that I see regularly for my healthcare<br>• A genetic counselor (a health professional trained in genetic diseases, particularly in returning and discussing implications of genetic testing results)<br>• A medical geneticist (a doctor who is specially trained in diagnosing and treating genetic diseases)<br>• A *BioMe* Biobank researcher or research coordinator<br>• Not applicable (I would not want to receive genetic research results)

Table S2. Demographic characteristics of Bio*Me* participants who were respondents of a return of genomic results preference survey (N = 72), and characteristics of all adult Bio*Me* participants enrolled at the time of the survey (N = 31,213).

Demographic characteristics (number of survey respondents)	Survey respondents N (%)	Bio*Me* participants N (%)	Chi-squared P value
Female (72)	43 (59.7)	18449 (59.1)	0.9
Age range (72)			<0.0001
18 to 30 years	27 (37.5)	2149 (6.9)	
31 to 50 years	16 (22.2)	8823 (28.3)	
51 to 70 years	26 (36.1)	13538 (43.4)	
71 years and over	3 (4.2)	6703 (21.5)	
Born in the U.S. (71)	62 (87.3)	19692 (63.1)	<0.0001
Self-reported ancestry (72)			0.07
African American/African	9 (12.5)	7670 (24.6)	
East/Southeast Asian	2 (2.8)	866 (2.8)	
Hispanic/Latinx	24 (33.3)	11072 (35.5)	
European	30 (41.7)	9459 (30.3)	
Native American, Other, or Multiple Selected	7 (9.7)	2146 (6.9)	
Jewish (70)	20 (28.6)	-	-
Identify as religious (69)	39 (56.5)	-	-
Have children (70)	26 (37.1)	-	-
Highest level of school completed or highest degree (70)		-	-
Less than high school degree	1 (1.4)		
High school degree or equivalent (e.g. GED)	4 (5.7)		
Some college but no degree	16 (22.9)		
Associate degree	4 (5.7)		
Bachelor degree	26 (37.1)		
Graduate degree	19 (27.1)		
Average household income (66)		-	-
Less than $20,000	9 (13.6)		
$20,000-$39,000	6 (9.1)		
$40,000-$59,000	14 (21.2)		
$60,000-$79,000	9 (13.6)		
$80,000-$149,000	10 (15.1)		
$150,000 or more	18 (27.2)		

Table S3. Clinically confirmed pathogenic, likely pathogenic, and downgraded variants in a pilot genomic screening program.

Gene	CHR:POS:REF:ALT	cDNA Position	Protein Position	# Heterozygous Carriers	Interpretation[a]
BRCA1	17:43057062:T:TG	c.5266dupC	p.Gln1756fs	2	Pathogenic
	17:43124027:ACT:A	c.68_69delAG	p.Glu23fs	4	Pathogenic
	17:43094472:C:T	c.1059G>A	p.Trp353Ter	1	Pathogenic
	17:43063917:A:C	c.5109T>G	p.Tyr1703Ter	1	Pathogenic
	17:43049191:TG:T	c.5335delC	p.Gln1779fs	1	Pathogenic
	17:43092615:TC:T	c.2915delG	p.Gly972fs	1	Pathogenic
	17:43091455:T:TGC	c.4074_4075dupGC	p.Gln1359fs	1	Likely pathogenic
BRCA2	13:32340128:C:T	c.5773C>T	p.Gln1925Ter	1	Pathogenic
	13:32338277:G:T	c.3922G>T	p.Glu1308Ter	2	Pathogenic
	13:32319298:G:T	c.289G>T	p.Glu97Ter	1	Pathogenic
	13:32380085:C:T	c.9196C>T	p.Gln3066Ter	1	Pathogenic
	13:32340300:GT:G	c.5946delT	p.Ser1982fs	3	Pathogenic
	13:32336781:T:G	c.2426T>G	p.Leu809Ter	1	Pathogenic
	13:32338783:CA:C	c.4429delA	p.Ile1477fs	1	Pathogenic
	13:32370955:G:A	c.8488-1G>A		1	Pathogenic
	13:32339489:G:T	c.5134G>T	p.Gly1712Ter	1	Pathogenic
	13:32340836:GACAA:G	c.6486_6489delACAA	p.Lys2162fs	1	Pathogenic
	13:32336684:G:GA	c.2330dupA	p.Asp777fs	1	Pathogenic
	13:32333282:G:T	c.1804G>T	p.Gly602Ter	1	Pathogenic
MLH1	3:37012098:C:T	c.676C>T	p.Arg226Ter	1	Pathogenic
MSH6	2:47799823:TC:T	c.1842delC	p.Cys615fs	1	Pathogenic
PMS2	7:6005918:C:A	c.137G>T	p.Ser46Ile	1	Pathogenic
	7:5986838:G:A	c.1927C>T	p.Gln643Ter	1	Pathogenic
	7:5986933:A:AT	c.1831dupA	p.Ile611fs	1	Pathogenic
	7:6003793:C:A	c.251-1G>T		1	Likely pathogenic
APOB	2:21006288:C:T	c.10580G>A	p.Arg3527Gln	1	Pathogenic
	2:21002392:CAT:C	c.13028_13029delAT	p.Tyr4343fs	2	Uncertain
	2:21006128:G:C	c.10740C>G	p.Asn3580Lys	2	Uncertain
	2:21005155:TG:T	c.11712delC	p.Asn3904Lysfs	1	Pathogenic[b]
LDLR	19:11116198:A:G	c.1691A>G	p.Asn564Ser	1	Likely pathogenic
	19:11105441:G:A	c.535G>A	p.Glu179Lys	1	Likely pathogenic
	19:11120143:C:T	c.1897C>T	p.Arg633Cys	1	Pathogenic
	19:11123263:C:T	c.2230C>T	p.Arg744Ter	1	Pathogenic
	19:11105567:G:A	c.661G>A	p.Asp221Asn	1	Pathogenic
	19:11116153:G:A	c.1646G>A	p.Gly549Asp	1	Pathogenic
	19:11113608:G:A	c.1432G>A	p.Gly478Arg	1	Likely pathogenic
	19:11113343:G:A	c.1252G>A	p.Glu418Lys	1	Uncertain
	19:11120106:G:T	c.1860G>T	p.Trp620Cys	1	Uncertain
	19:11120188:T:G	c.1942T>G	p.Ser648Ala	2	Uncertain
	19:11131285:A:G	c.2552A>G	p.Gln851Arg	1	Uncertain
	19:11105507:G:A	c.601G>A	p.Glu201Lys	1	Uncertain
	19:11105572:C:T	c.666C>T	p.D222=	1	Uncertain
TTR	18:31598655:G:A	c.424G>A	p.Val142Ile	33	Pathogenic
	18:31592974:G:A	c.148G>A	p.Val50Met	1	Pathogenic

[a] Only pathogenic and likely pathogenic variants associated with conditions included in the pilot genomic screening program were disclosed to consenting participants.
[b] This *APOB* variant is associated with hypobetalipoproteinemia.
cDNA and protein position provided for NM_007294.3 (*BRCA1*), NM_000059.3 (*BRCA2*), NM_000249.3 (*MLH1*), NM_000179.2 (*MSH6*), NM_000535.5 (*PMS2*), NM_000384.2 (*APOB*), NM_000527.4 (*LDLR*), and NM_000371.3 (*TTR*); Human reference genome build 38 (GRCh38).

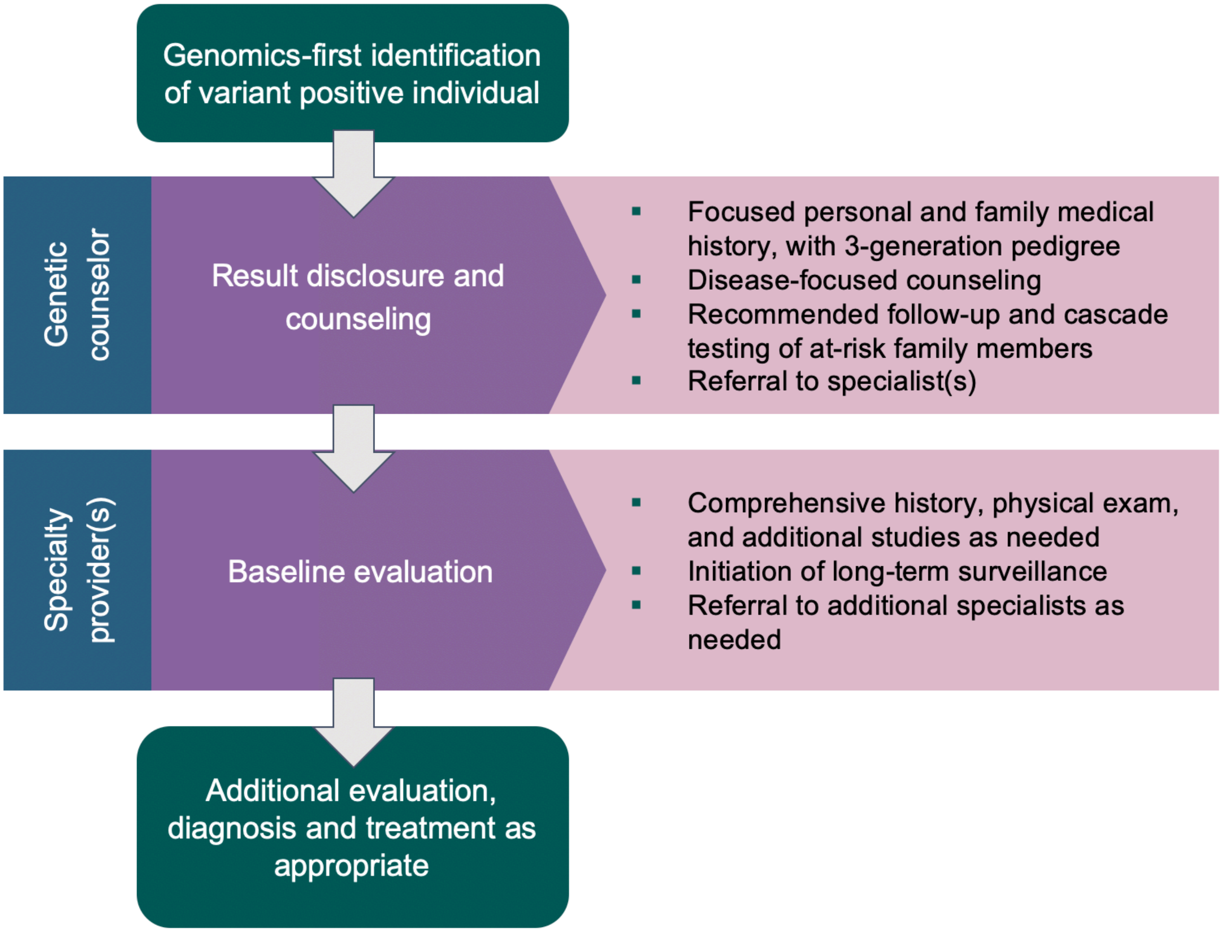

Fig. S1. A model for returning genomic results to biobank research participants without prior knowledge of genomic risk. After genomics-first identification of variant positive individuals, they receive genetic counseling and are then referred to specialty provider(s) for further evaluation and initiation of risk management.

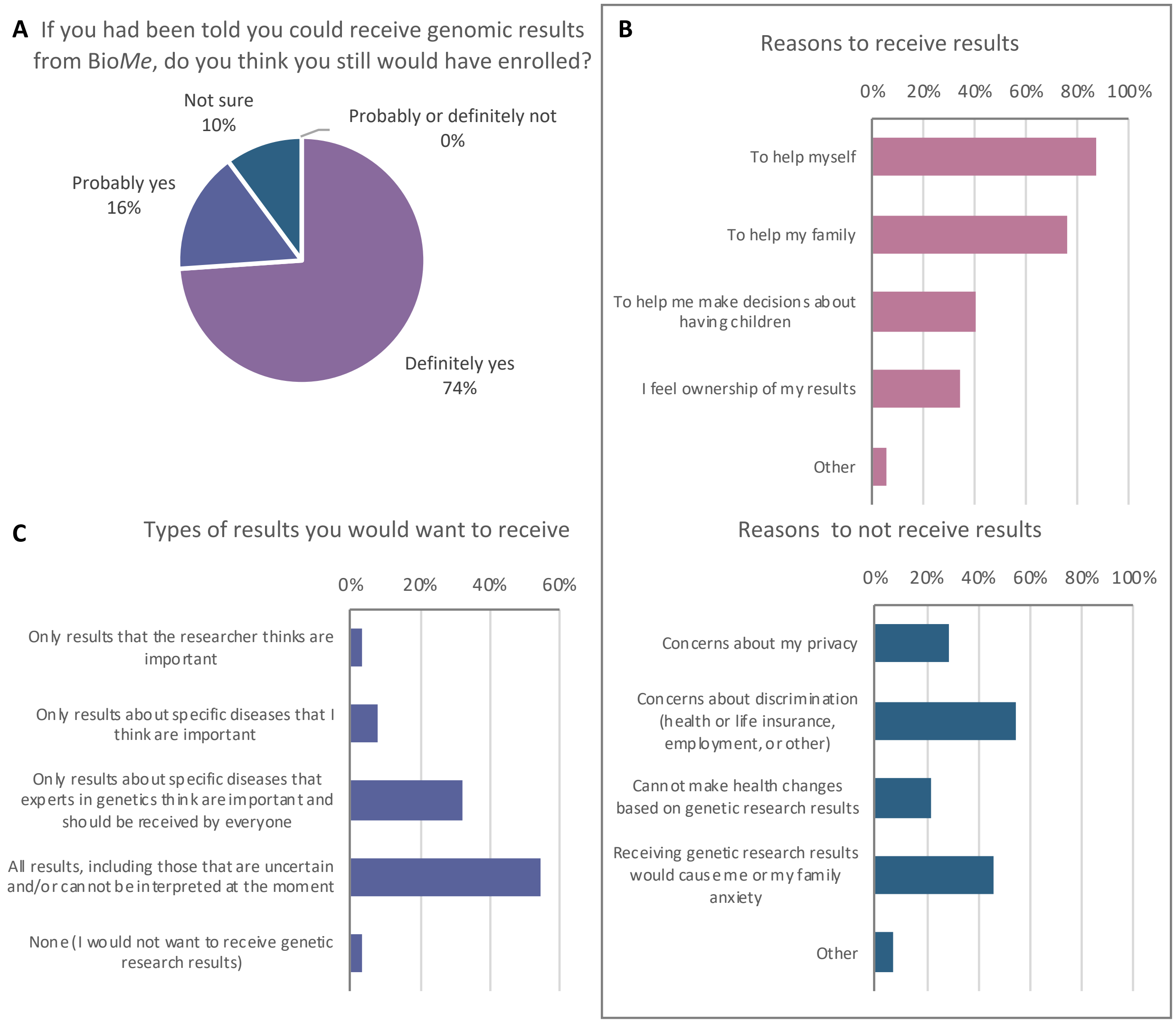

Fig. S2. Pre-pilot survey to understand Bio*Me* participants' (N = 72) preferences regarding the hypothetical return of results. A) Decision to enroll into biobank research if the program returned genomic results. **B)** Selected reasons for receiving or not receiving genomic results. **C)** Selected categories of genomic results participants would want to receive.
